# Systemic treatment of patients with locally advanced or metastatic cholangiocarcinoma – an Austrian expert consensus statement

**DOI:** 10.3389/fonc.2023.1225154

**Published:** 2023-08-30

**Authors:** Hossein Taghizadeh, Angela Djanani, Wolfgang Eisterer, Armin Gerger, Birgit Gruenberger, Thomas Gruenberger, Holger Rumpold, Lukas Weiss, Thomas Winder, Ewald Wöll, Gerald W. Prager

**Affiliations:** ^1^ Department of Internal Medicine I, University Hospital St. Pölten, St. Pölten, Austria; ^2^ Karl Landsteiner University of Health Sciences, Krems, Austria; ^3^ Clinical Division of Gastroenterology, Hepatology and Metabolism, Department of Internal Medicine, Medical University of Innsbruck, Innsbruck, Austria; ^4^ Department of Internal Medicine, Klagenfurt Hospital, Klagenfurt am Wörthersee, Austria; ^5^ Department of Internal Medicine, Clinical Division of Oncology, Medical University of Graz, Graz, Austria; ^6^ Department of Internal Medicine and Hematology and Internal Oncology, Landesklinikum Wiener Neustadt, Wiener Neustadt, Austria; ^7^ Department of Surgery, Clinic Favoriten, Hepatopancreatobiliary Center (HPB) Center, Health Network Vienna, and Sigmund Freud Private University, Vienna, Austria; ^8^ Visceral Oncology Center, Ordensklinikum Linz, Linz, Austria; ^9^ Department of Internal Medicine III, Paracelsus Medical University, Salzburg, Austria; ^10^ Department of Internal Medicine II, Hospital Feldkirch, Feldkirch, Austria; ^11^ Department of Internal Medicine, Saint Vincent Hospital Zams, Zams, Austria; ^12^ Department of Medicine I, Clinical Division of Oncology, Medical University of Vienna, Vienna, Austria

**Keywords:** biliary tract cancer (BTC), cholangiocarcinoma, molecular profiling, targeted therapy, chemotherapy

## Abstract

Locally advanced or metastatic cholangiocarcinoma is an aggressive carcinoma with a dismal prognosis. For the first-line treatment of locally advanced or metastatic cholangiocarcinoma, cisplatin/gemcitabine has been the standard of care for more than 10 years. Its combination with the immune checkpoint inhibitor durvalumab resulted in an efficiency improvement in the phase III setting. Regarding the use of chemotherapy in the second line, positive phase III data could only be generated for FOLFOX. The evidence base for nanoliposomal irinotecan (Nal-IRI) plus 5-fluorouracil (5-FU) and leucovorin (LV) is contradictory. After the failure of first-line treatment, targeted therapies can be offered if the molecular targets microsatellite instability-high (MSI-H), IDH1, FGFR2, BRAF V600E, and NTRK are detected. These targeted agents are generally preferable to second-line chemotherapy. Broad molecular testing should be performed, preferably from tumor tissue, at the initiation of first-line therapy to timely identify potential molecular targets.

## Introduction

The term “cholangiocarcinoma” (CCA) comprises a group of heterogeneous malignant tumors arising at any point of the biliary tree. Three subtypes of CCA are distinguished according to the anatomical site of origin: intrahepatic, perihilar, and distal (1, 2). Although CCA is a rare cancer, epidemiological data suggest an increasing global burden over the last decades, with rising annual rates of incidence (0.3–6/100,000 inhabitants) and mortality (1–6/100,000 inhabitants) (1, 3). Most patients present with advanced disease because CCAs are usually asymptomatic in the early stages (1, 4). Despite increased awareness and improved therapies, patient prognosis is still poor. The 5-year survival rates range between 7 and 20%, and recurrence is likely after resection (5–12).

At present, the systemic treatment landscape is expanding, while the currently available options leave room for discussion regarding the ideal choice and sequence of therapies. Therefore, Austrian experts in the field of medical oncology and liver surgery convened on 9^th^ October 2022, to reach a consensus on the systemic treatment of non-resectable, locally advanced, or metastatic CCA.

## First-line treatment

The phase III ABC-02 trial published in 2010 established cisplatin plus gemcitabine as the first-line treatment standard in patients with advanced biliary tract cancer (13). Compared to the single agent gemcitabine, the platinum-based combination improved median overall survival (OS) by 3.6 months (11.7 vs. 8.1 months), which translated into a 36% mortality reduction (HR: 0.64; p < 0.001). All subgroups derived OS benefits.

This long-lasting standard regimen was recently augmented with the anti-PD-L1 antibody durvalumab based on the phase III TOPAZ-1 trial that explored its addition (14). The OS advantage provided by durvalumab plus cisplatin/gemcitabine vs. cisplatin/gemcitabine alone was statistically significant, even though the relative risk reduction did not exceed 20% (median OS: 12.9 vs. 11.3 months; HR: 0.76 [CI: 0.64–0.91]). In the experimental arm, the Kaplan–Meier curve plateaued beyond 18 months, which gave rise to 2-year OS rates of 23.6 vs. 11.5%. This indicates that a subgroup of patients derives sustained benefit from the triple combination. Prolonged survival on the combination is most likely among fit patients.

Due to the long-term effect observed in TOPAZ-1, durvalumab plus cisplatin/gemcitabine has been endowed with a 4-point score according to the ESMO-Magnitude of Clinical Benefit Scale (MCBS) (15). However, it must be noted that the regimen would have been given a score of 1 without the ≥ 10% increase in 2-year survival. Considering the limited statistical power of this comparison based on a total of 13 patients across the 2 study arms at 24 months, this “upgrade” according to the ESMO-MCBS scoring appears at least debatable. Nevertheless, the TOPAZ-1 trial has introduced an evidence-based, potentially highly beneficial treatment option after a decade-long standstill in first-line strategies in the advanced CCA setting. Based on the TOPAZ-1 data, durvalumab plus cisplatin/gemcitabine has been approved by the US Food and Drug Administration and recently by the European Medicines Agency for the first-line treatment of biliary tract cancer.

**Figure 1:**
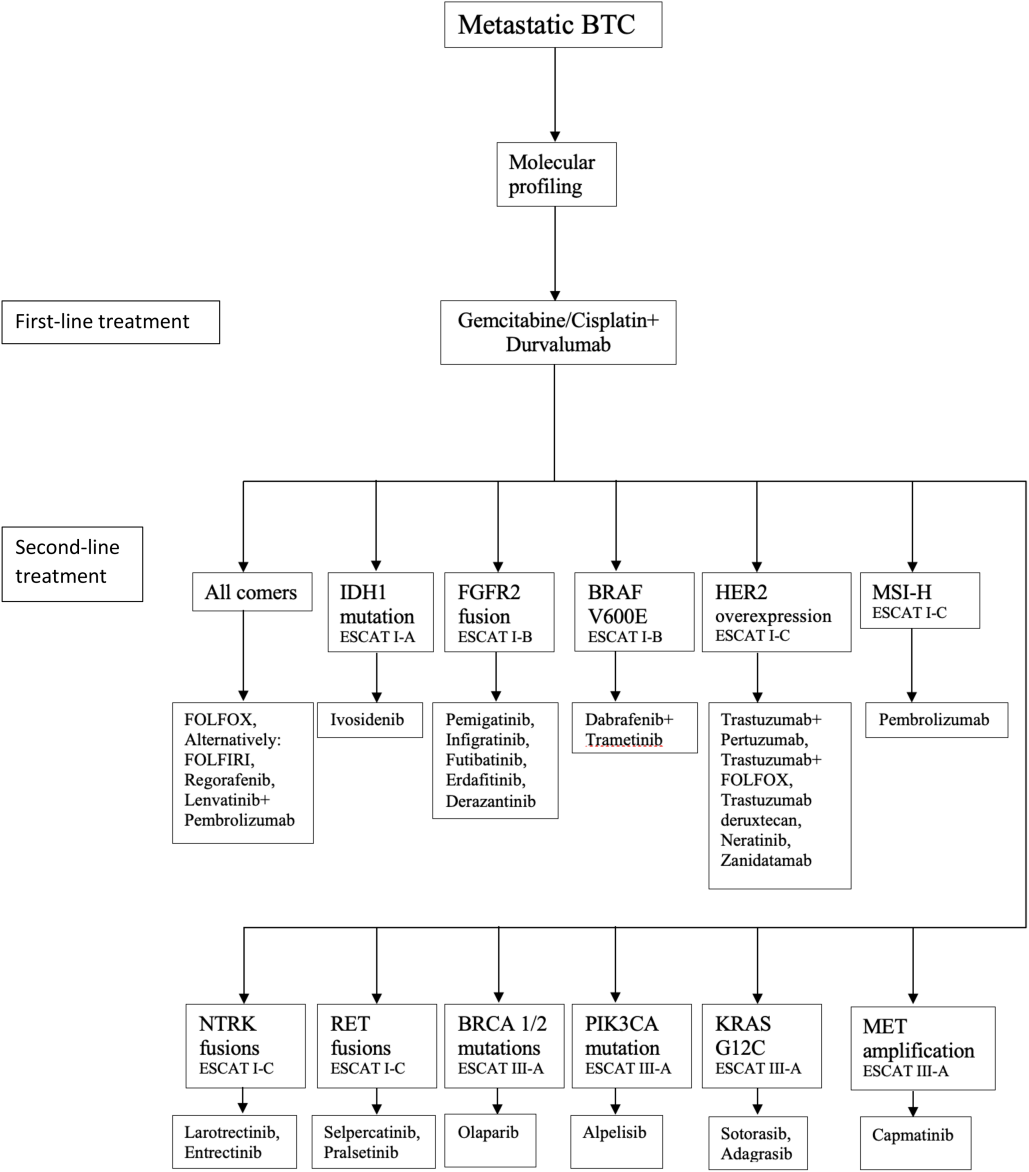
Treatment algorithm for biliary tract cancer – modified ESMO Clinical Practice Guideline for advanced biliary tract cancer (16).

Another factor favoring the immunotherapy-based approach is the possibility of durvalumab maintenance after discontinuation of the cisplatin/gemcitabine backbone. This offers increased tolerability compared to continued administration of the platinum-based regimen, whose prolonged use inevitably evokes complications such as neuropathy. In the TOPAZ-1 study, durvalumab use did not add to the overall toxicity, and the rates of grade 3/4 adverse events were similar across the 2 treatment arms (15). The ESMO Clinical Practice Guideline for the diagnosis and treatment of biliary tract cancer recommends cisplatin/gemcitabine in locally advanced or metastatic biliary tract cancer, while the addition of durvalumab can be considered (**Figure 1**) (16).

All patients should be re-evaluated regarding potential surgical or locally ablative interventions at regular intervals. It is important for tumor boards to include surgeons specialized in liver surgery, particularly for the assessment of resectability, and to repeat multidisciplinary team discussions 2–3 months after treatment initiation.

- The addition of durvalumab to first-line cisplatin/gemcitabine should be considered in patients who can be assumed to experience long-term overall survival benefits, that is, individuals with ECOG performance status scores of 0 or 1 who are eligible for doublet chemotherapy and have no contraindications to immune checkpoint inhibition.
- In patients who have at least achieved disease stabilization with cisplatin/gemcitabine plus durvalumab, durvalumab can be continued as single-agent maintenance therapy after 6 months (i.e., 8 cycles) of combined treatment.
- The reinduction of cisplatin/gemcitabine can be considered upon progression after a chemotherapy break of at least 6 months.
- In patients with locally advanced disease who are candidates for tumor resection, response evaluation to achieve resectability is recommended at 2-month intervals. Restaging in the metastatic setting, on the other hand, should be performed every 10–12 weeks.

## Second and later lines

### Chemotherapy

In the pretreatment setting, the ABC-06 trial is the only positive phase III study conducted to date (17). ABC-06 demonstrated a significant OS benefit of FOLFOX compared to active symptom control that translated into a 31% mortality reduction (median OS: 6.2 vs. 5.3 months; HR: 0.69; p = 0.031).

The Korean phase IIB NIFTY trial established nanoliposomal irinotecan (nal-IRI) plus fluorouracil (5-FU) and leucovorin (LV) as an alternative second-line regimen (18). Here, progression-free survival (PFS) was significantly improved compared to that of 5-FU/LV alone (7.1 vs. 1.4 months; HR: 0.56; p = 0.0019), as was OS (8.6 vs. 5.5 months; HR: 0.68; p = 0.0349). Each treatment arm contained up to 90 patients. However, these findings were not corroborated by the German NALIRICC (AIO-HEP-0116) study that included approximately 50 patients in each arm (19). Nal-IRI plus 5-FU/LV, as compared to 5-FU/LV, did not prolong PFS (HR: 0.867) or OS (HR: 1.082), while giving rise to an unexpectedly high adverse event rate.

These findings notwithstanding, nal-IRI plus 5-FU/LV is generally preferred over FOLFOX in Austrian centers and has demonstrated activity in clinical practice (20). Given the controversial data, it appears advisable to shorten the intervals between response assessments.

In cases where nal-IRI cannot be used, palliative therapy with lenvatinib combined with pembrolizumab or regorafenib may be considered for patients with ECOG 0-1 (21, 22).

- Second-line administration of FOLFOX is recommended in patients without targetable driver mutations based on phase III evidence.
- Nal-IRI plus 5-FU/LV represents an alternative option despite controversial phase II data. Early response evaluation after 2 months is recommended.
- The treatment selection should be based on factors such as performance status and toxicities of previous therapies (e.g., neuropathy).

### Targeted treatment

The molecular characterization of CCA has revealed several targetable driver aberrations and a growing array of targeted therapies is being established in routine clinical treatment. The *FGFR2* inhibitor pemigatinib has been approved for use in pretreated patients with *FGFR2* fusions or rearrangements based on the phase II FIGHT-202 trial, which showed an overall response rate of 37% and a disease control rate of 82% (23). Median PFS and OS were 7.0 and 17.5 months, respectively.

In patients with somatic *IDH1* mutations, the phase III ClarIDHy study revealed the superiority of the *IDH1* inhibitor ivosidenib over placebo regarding PFS (2.7 vs. 1.4 months; HR: 0.37; p < 0.0001) and OS (10.3 vs. 5.1 months after adjustment for crossover; HR: 0.49; p < 0.0001) (24, 25). Disease control was achieved in 53.2 vs. 27.9% of cases (24).

The single-arm phase II ROAR basket trial conducted on patients with rare tumor types showed promising activity of the *BRAF* inhibitor dabrafenib and the MEK inhibitor trametinib in 33 patients with *BRAF* ^V600E^-mutated biliary tract cancer (26). The response rate was 41%, and median PFS and OS were 7.2 and 11.3 months, respectively.

Moreover, the PD-1 inhibitor pembrolizumab has been licensed for use in the setting of previously treated microsatellite instability-high (MSI-H) or mismatch-repair-deficient biliary cancer.

Patients with *NTRK*-positive CCA can be treated with the *NTRK* inhibitors larotrectinib or entrectinib, which have received tumor-agnostic approval for advanced solid tumors harboring *NTRK* fusions.

The recommendations for the use of next-generation sequencing for patients with metastatic cancers are based on the ESMO Scale for Clinical Actionability of molecular Targets (ESCAT) (27). *IDH1* mutations, *FGFR2* fusions, MSI-H, *NTRK* fusions, *BRAF* ^V600E^ mutations and *ERBB2 (HER2)* have been classified as level I and three other aberrations as level III according to the most recent ESMO Guideline for Biliary Tract Cancer (**Table 1**) (16). At present, no agents are approved for the treatment of patients with level II and III alterations in CCA.

**Table 1:** ESCAT levels of genomic alterations according to the ESMO Clinical Practice Guideline for biliary tract cancer (16).

Gene	Alteration	Prevalence	ESCAT
*IDH1*	Mutations	20%	IA
*FGFR2*	Fusions	15%	IB
*BRAF* ^V600E^	Mutations	5%	IB
	MSI-H/dMMR	2%	IC
*NTRK*	Fusions	2%	IC
*ERBB2*	Amplifications Mutations	10% 2%	IC –
*PIK3CA*	Mutations	7%	IIIA
*BRCA 1/2*	Mutations	3%	IIIA
*MET*	Amplifications	2%	IIIA

Considering the ongoing research efforts in the field of targeted agents, comprehensive testing based on large panels covering driver aberrations beyond those listed by the ESMO Precision Medicine Working Group is encouraged with a view toward patient inclusion in future clinical studies. In addition, *BRCA* 1/2 testing can identify families with an increased risk of other cancers.

- Molecular testing of a broad range of targets is recommended prior to the initiation of first-line systemic treatment.
- Whenever possible, testing should be performed based on tumor tissue.
- As tissue can be difficult to obtain in the advanced setting, liquid biopsy constitutes a valid alternative. Negative liquid biopsy results do not completely preclude the presence of driver aberrations and should be confirmed if tissue becomes available later.
- Targeted treatment should be preferred over second-line chemotherapy in patients with ESCAT level I (and II) alterations.
- Patients with level (II and) III targets, in whom evidence-based regimens have been exhausted, should be discussed by the molecular tumor board.
- No standard third-line treatments have been defined to date. Oxaliplatin- or irinotecan-based chemotherapy can be administered upon progression following targeted treatment.

## Conclusion

The recommendations on the systemic treatment of locally advanced or metastatic CCA summarized in this paper mirror the availability of therapies and reimbursement situation in Austria as of autumn 2022 amidst a changing treatment landscape. Data have recently been generated regarding the addition of first-line durvalumab to the chemotherapeutic standard, and despite their limited statistical power, the introduction of immunotherapy represents a potential improvement for certain patients. In the second-line setting, targeted treatment based on potential molecular aberrations is the preferred option.

It is strongly recommended to extend molecular testing beyond the established genomic aberrations, as CCA patients—who should be treated at specialized centers as a matter of principle—might get the opportunity to enter clinical trials investigating new compounds. Innovative agents as well as drugs that have already been implemented for other cancers might become accessible over the coming years, thus redefining the current algorithms and taking the systemic treatment of CCA patients to the next level. For patients without druggable targets, FOLFOX is a potential second-line treatment option with a high level of evidence based on a positive phase III clinical trial (17). The use of liposomal irinotecan plus chemotherapy is controversial, although this treatment has shown efficacy in clinical trials and daily practice and thus might be considered a valid second-line treatment option.

## Data availability statement

The original contributions presented in the study are included in the article/supplementary material. Further inquiries can be directed to the corresponding author.

## Author contributions

All authors listed have made a substantial, direct, and intellectual contribution to the work and approved it for publication.
